# Special Issue on Networks-on-Chip Again on the Rise: From Emerging Applications to Emerging Technologies

**DOI:** 10.3390/mi12121570

**Published:** 2021-12-17

**Authors:** Davide Bertozzi, José L. Abellán, Mahdi Nikdast

**Affiliations:** 1 Engineering Department, University of Ferrara, 44122 Ferrara, Italy; 2 Computer Science and Engineering Department, Universidad Católica de Murcia (UCAM), 30107 Murcia, Spain; jlabellan@ucam.edu; 3 Electrical and Computer Engineering Department, Colorado State University, Fort Collins, CO 80523, USA; mahdi.nikdast@colostate.edu

Twenty years after the advent of interconnection networks to tackle the on-chip communication bottleneck [1], integrated computing platforms are again interconnect-dominated. First, the future of computing beyond Moore’s law and Dennard scaling is moving towards Systems-in-Package (SiP) based computing platforms that leverage advanced integration technologies such as 2.5D or 3D stacking [2]. In this context, emerging interconnect technologies aim at sustaining the levels of system integration by delivering performance and power metrics that are out-of-reach for conventional electronics [3]. Second, the advent and consolidation of data-intensive applications from artificial intelligence and big data analytics is putting unprecedented pressure on interconnection fabrics at each layer of the compute hierarchy [4]. Third, the emergence of novel computing paradigms consisting of domain-specific accelerators, optical computing, quantum computing or neuromorphic computing can truly make an impact provided matching interconnect architectures and technologies are developed [5]. Fourth, the increased usage of networks-on-chip (NoCs) and their distributed nature across integrated circuits have made them a focal point of potential security attacks [6].

In this context, network-on-chip research is again on the rise, thereby generating a second wave of “communication-centric designs.” Unlike the early days, when the first NoC architectures were designed, the same concept nowadays holds at a radically different abstraction level: it refers to novel system-level design paradigms leveraging the enabling features of communication architectures and emerging technologies. Thus, NoC research is even more exciting than it used to be because researchers have to break barriers between disciplines and take a fundamental cross-layer approach to design and optimization of more complex NoCs.

This Special Issue provides an overview of the ongoing research efforts that are trying to bring on-chip interconnection networks into new ground. It consists of eight papers covering some of the most sensitive aspects of the ongoing network-on-chip evolution, as hereafter illustrated.

(1) Most of the papers deal with the increasing complexity of efficient network-on-chip management in the presence of multi-programmed platforms (where several applications are consolidated onto a unified parallel hardware platform) and/or data-intensive applications, raising unprecedented mapping, congestion control and bandwidth allocation concerns. 

F. Ge et al. [7] target the simultaneous deployment of multiple applications onto the same NoC-based system. Among other application domains, this scenario is of interest for the Edge computing environment, where multiple Internet-of-Thing services contend for shared resources on cost-constrained computational platforms. A genetic algorithm is used for application mapping into potential regions, while a simulated annealing algorithm generates the optimal placement for the multi-application mapping regions.

The delivery of fine-grained bandwidth allocation through suitable arbitration mechanisms is the focus of S. Ibarra-Delgado et al. [8], aiming at the fulfillment of QoS requirements of communication actors in a bus or NoC. The proposed arbitration policy is based on opportunistic access and supervised depth techniques that, when combined, outperform traditional Lottery, TDMA and Weighted Round-Robin schemes in terms of bandwidth control capability.

Regional congestion awareness routing algorithms have shown great potential in improving NoC performance under traffic pressure. However, they incur significant queuing latency. The work of J. Fang et al. [9] proposes a novel routing strategy that partitions the network into two areas (edge area and central area) based on node priorities and handles routing inside each of them differently for the sake of lower latency and higher saturation throughput over conventional approaches. 

Finally, the survey from J.R. Gomez-Rodriguez et al. [10] reviews state-of-the-art on Software-Defined Networks-on-Chip (SDNoCs), which investigates the successful application of Software-Defined Networking to elegantly solve computer networks’ management problems for the NoC domain. Expected benefits include higher flexibility for runtime and self-adaptive network management and a reduced hardware complexity for routers, although the literature is currently lagging far behind in architecture layering for interoperability.

(2) Another group of papers tackles some of the challenges for porting the chip networking paradigm onto a different technology substrate with respect to conventional digital electronics. The focus is on silicon nanophotonic networks, fully replacing their electronic counterparts or complementing them through hybrid interconnection architectures. Critical aspects of optical network-on-chip design include insertion loss and crosstalk loss, which directly affect the overall power budget and signal-to-noise ratio of this emerging interconnect solution, and hence its practical feasibility. 

Optical NoCs (ONoCs) require rethinking of routing strategies, since optical routing frameworks should be tailored to network topologies, router structures and photonic switching mechanisms. The work from Y.L. Zheng et al. [11] adopts the Dijkstra algorithm to realize adaptive routing with minimum transmission loss of links and reduce the output power of link transmitters in mesh-based ONoCs with respect to traditional dimension-order routing. The routing algorithm proposed by X.P. Yang et al. [12] selects the routing paths with minimum power loss, while at the same time ensuring that the routing paths have approximately the optimal optical signal-to-noise ratio (OSNR). The algorithm relies on models for power loss, crosstalk loss and all-pass optical router operation. 

Cleary, minimization of insertion loss and crosstalk noise is at the core of each ONoC optimization framework. For this reason, the work from T. Song et al. [13] proposes a network-level analysis method for optical losses that aims to be a reliable theoretical basis and technical support for an efficient ONoC design. 

(3) Last but not least, this Special Issue hosts one paper that is only indirectly related to network-on-chip design, yet with potential impact on the community. In fact, the work of J. Liu et al. [14] employs stochastic computing to design a hardware pseudo-random number generator with low hardware utilization, which indeed saves 89% of hardware resources compared to conventional approaches. The importance of such hardware devices is paramount for designing future secure communication protocols, but is relevant for other domains as well, including cryptography, digital signatures and image authentication watermarks.

In conclusion, this Special Issue provides valuable insights into promising directions for future NoC research. We would like to take this opportunity to thank all the authors for submitting their papers and all the reviewers for dedicating their time and helping to improve the quality of the submitted papers.

## References

[1] Benini L., De Micheli G. (2002). Networks on chips: A new SoC paradigm. Computer.

[2] Franzon P.D., Marinissen E.J., Bakir M.S. (2019). Handbook of 3D Integration, Volume 4: Design, Test, and Thermal Management.

[3] Karkar A., Mak T., Tong K., Yakovlev A. (2016). A Survey of Emerging Interconnects for On-Chip Efficient Multicast and Broadcast in Many-Cores. IEEE Circuits Syst. Mag..

[4] Bernabé S., Wilmart Q., Hasharoni K., Hassan K., Thonnart Y., Tissier P., Désières Y., Olivier S., Tekin T., Szelag B. (2021). Silicon photonics for terabit/s communication in data centers and exascale computers. Solid-State Electron..

[5] Moradi S., Qiao N., Stefanini F., Indiveri G. (2018). A Scalable Multicore Architecture with Heterogeneous Memory Structures for Dynamic Neuromorphic Asynchronous Processors (DYNAPs). IEEE Trans. Biomed. Circuits Syst..

[6] Mishra P., Charles S. (2021). Network-on-Chip Security and Privacy.

[7] Fen G., Cui C., Zhou F., Wu N. (2021). A Multi-Phase Based Multi-Application Mapping Approach for Many-Core Networks-on-Chip. Micromachines.

[8] Salvador I., Sandoval-Arechiga R., Gómez-Rodríguez J.R., Ortíz-López M., Brox M. (2020). A Bandwidth Control Arbitration for SoC Interconnections Performing Applications with Task Dependencies. Micromachines.

[9] Juan F., Zhang D., Li X. (2020). ParRouting: An Efficient Area Partition-Based Congestion-Aware Routing Algorithm for NoCs. Micromachines.

[10] Gomez-Rodriguez J.R., Sandoval-Arechiga R., Ibarra-Delgado S., Rodriguez-Abdala V.I., Vazquez-Avila J.L., Parra-Michel R. (2021). A Survey of Software-Defined Networks-on-Chip: Motivations, Challenges and Opportunities. Micromachines.

[11] Yan-Li Z., Song T., Chai J., Yang X., Yu M., Zhu Y., Liu Y., Xie Y. (2021). Exploring a New Adaptive Routing Based on the Dijkstra Algorithm in Optical Networks-on-Chip. Micromachines.

[12] Xiao-Ping Y., Song T., Ye Y., Liu B., Yan H., Zhu Y., Zheng Y., Liu Y., Xie Y. (2020). A Novel Algorithm for Routing Paths Selection in Mesh-Based Optical Networks-on-Chips. Micromachines.

[13] Tingting S., Xie Y., Ye Y., Wang S., Du Y. (2020). Crosstalk Analysis and Performance Evaluation for Torus-Based Optical Networks-on-Chip Using WDM. Micromachines.

[14] Junxiu L., Liang Z., Luo Y., Cao L., Zhang S., Wang Y., Yang S. (2021). A Hardware Pseudo-Random Number Generator Using Stochastic Computing and Logistic Map. Micromachines.

